# Matching of array CGH and gene expression microarray features for the purpose of integrative genomic analyses

**DOI:** 10.1186/1471-2105-13-80

**Published:** 2012-05-04

**Authors:** Wessel N van Wieringen, Kristian Unger, Gwenaël GR Leday, Oscar Krijgsman, Renée X de Menezes, Bauke Ylstra, Mark A van de Wiel

**Affiliations:** 1 Department of Epidemiology and Biostatistics, VU University Medical Center, P.O. Box 7057, Amsterdam, 1007, MB, The Netherlands; 2 Department of Mathematics, VU University Amsterdam, De Boelelaan 1081a, Amsterdam, 1081, HV, The Netherlands; 3 Research Unit of Radiation Cytogenetics, Helmholtz Zentrum München, Ingolstädter-Landstrasse 1, Neuherberg, 85764, Germany; 4 Department of Pathology, VU University Medical Center, P.O. Box 7057, Amsterdam, 1007, MB, The Netherlands

## Abstract

**Background:**

An increasing number of genomic studies interrogating more than one molecular level is published. Bioinformatics follows biological practice, and recent years have seen a surge in methodology for the integrative analysis of genomic data. Often such analyses require knowledge of which elements of one platform link to those of another. Although important, many integrative analyses do not or insufficiently detail the matching of the platforms.

**Results:**

We describe, illustrate and discuss six matching procedures. They are implemented in the R-package sigaR (available from Bioconductor). The principles underlying the presented matching procedures are generic, and can be combined to form new matching approaches or be applied to the matching of other platforms. Illustration of the matching procedures on a variety of data sets reveals how the procedures differ in the use of the available data, and may even lead to different results for individual genes.

**Conclusions:**

Matching of data from multiple genomics platforms is an important preprocessing step for many integrative bioinformatic analysis, for which we present six generic procedures, both old and new. They have been implemented in the R-package sigaR, available from Bioconductor.

## Background

DNA copy number aberrations abound in the cancer cell. The location, size and direction of these aberrations vary between cancers of different tissues, between cancers of the same tissue, and may even exhibit heterogeneity among cells originating from the same tumor [1]. The DNA copy number aberrations often span a genomic region encoding one or multiple transcripts. The expression levels of such transcripts may be affected (in a variety of ways) by the abnormal gene dosage. In turn, the affected transcription levels may have consequences for the cancer cell.

The elucidation of the relationship between DNA copy number aberrations and mRNA (and microRNA) transcript levels is key to enhance our understanding of the regulatory mechanism of the cancer cell. To this end, oncogenomic studies profile both the genome and transcriptome of a large number of tumors of the same tissue, of which [2,3] are the first examples. The present day shows studies involving many more samples, which are profiled on increasingly higher resolution platforms (e.g., [4-8]). Bioinformatics follows biological practice. First, only few, relatively simple procedures for the integrative analysis of DNA copy number and gene expression data appeared (e.g., [9-11]). The last few years, however, have seen a surge in more sophisticated methodology addressing a wide range of biological questions involving the two molecular levels (e.g., [12-24]).

In order to investigate the *cis*- (and *trans*-) effect of DNA copy number changes on expression, we need, for each gene measured, its corresponding copy number information. If both profiles are generated on the same platform this is immediate. Pollack *et al*. [25] and Van de IJssel *et al*. [26] showed that DNA copy number and gene expression data can indeed be generated on the same platform, cDNA and oligo, respectively. If the data stem from different platforms, features from both platforms need to be matched. This matching requires to resolve two key problems:

• Search for DNA copy number features that are (in some sense) close to gene expression features.

• Summarize the data of the DNA copy number features that match to the same gene expression feature.

 Matching is important for the downstream integrative analyses. Although important, many integrative analyses of DNA copy number and gene expression data do not or insufficiently detail the matching of the two platforms. In this paper we present the matching procedures that we have come across and developed. They are implemented in the R-package sigaR (available from Bioconductor). The principles underlying the presented matching procedure are generic, and can be combined to form new matching approaches, or be applied to the matching of other platforms (facilitated by the implementation of the distanceAny and overlapAny procedures in sigaR). We apply the matching procedures to five data sets. This illustrates them any consequences of employing a particular matching procedure, and suggests ways of making an informed choice on the method best applied to the data set at hand.

## Implementation

Six procedures and three extensions implemented in the R-package sigaR for matching the features of a DNA copy number and gene expression array are outlined. Starting point of each procedure are two R-objects: one of the cghCall-class and one of the ExpressionSet-object as defined in the packages CGHbase and Biobase, both available via Bioconductor. The cghCall object contains the DNA copy number data (in its various forms [27]: normalized, segmented, and called data) and annotation information of the features of the array (e.g., label, chromosome, start and end base pair information of the features). The ExpressionSet object comprises the preprocessed gene expression data plus annotation information of the array on which the data were generated. Below we describe each matching procedure implemented in sigaR (with one exception, see later) in detail, and discuss its pro’s and cons. Example R code for each matching procedure using sigaR is provided in Appendix B.

The number of conceivable matching procedures is possibly infinite, so we do not claim to provide an exhaustive list. Instead the focus is on procedures that address the key problems in the matching of features from different platforms. The principles underlying the described matching procedures may be varied upon and combined endlessly to generate new matching procedures. Also, many of these principles apply to the matching of platforms interrogating different molecular levels, or other techniques as massive parallel sequencing (MPS).

### Label

**Figure 1:**
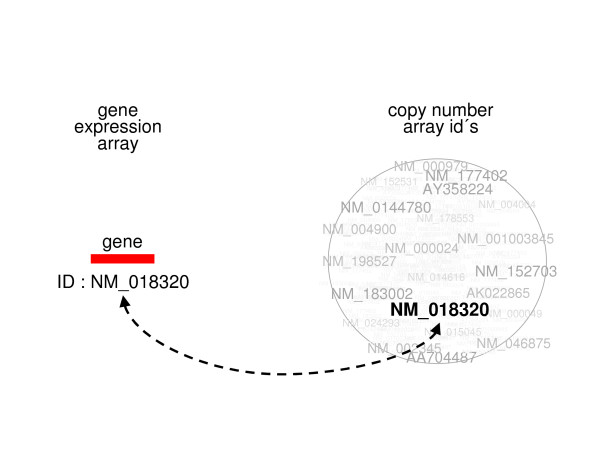
**Label matching: an ID is sought within a large set of IDs.** The dashed arrow indicates that the gene’s ID has been found in the pile of DNA copy number IDs.

The first matching procedure uses the feature labels (e.g., manufacturer IDs) of both arrays. Both sets of labels are mapped to a common descriptor set, e.g., the gene symbol. These maps are exploited to link the features of the two platforms, and features of both platforms are matched if they map to the same common descriptor. See Lo *et al*. [28] for an application of this procedure. Table 1 describes the procedure algorithmically, while it is depicted visually in Figure 1.

**Table 1:** Label matching

*Step 1*
Map the manufacturer’s IDs of both platforms to that of a common reference set of IDs.
*Step 2*
For each gene on the expression array, find its ID within the set of reference IDs of DNA copy number array features.
*Step 3*
Assign the DNA copy number data of the matched feature to the gene.

The label matching procedure depends heavily on the quality of the maps to the common descriptor set. In particular, a feature of the DNA copy number array located on one chromosome might – in principle – be matched to a gene expression element located on another chromosome. Also, many features of both arrays may map to one descriptor, leading to non-unique matching.

Note that the label matching procedure is not implemented in the sigaR package. But labels can be matched directly using the match function available from the base of R.

### Distance

**Figure 2:**
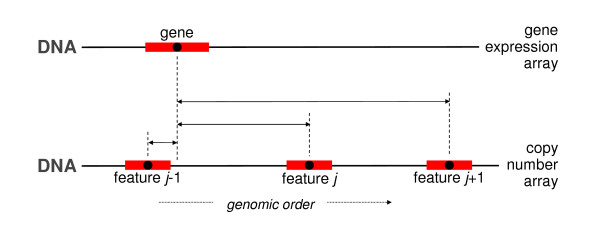
**Distance matching: the DNA copy number feature closest to the gene is sought.** The black points inside the red boxes represent the midpoints. Above, feature *j-1* clearly is closest to the gene.

As an alternative to the label information, features may also be matched on the basis of their genomic location. The first procedure we describe that does this defines a distance measure between the genomic locations of the two probe sequences. A gene expression feature is matched to the DNA copy number feature with the closest (mid-)base pair position. The distance matching procedure has, among others, been proposed by Van Wieringen *et al*. [11]. See Table 2 for an algorithmic description, with Figure 2 an illustration of the crucial step of the algorithm.

**Table 2:** Distance matching

*Step 1*
For all genes, calculate their midpoints (average of start and end base pair position). Do the same for the features on DNA copy number array.
*Step 2*
For each gene, calculate the distance between its midpoint and that of the DNA copy number array features (mapping to the same chromosome as the gene). See Figure 2.
*Step 3*
Assign to each gene the DNA copy number data of the feature with minimum distance between its midpoint and that of the gene.

Matching by distance may link two features that are considerably separated genomically. Then, the existence of a *cis*-effect of one feature’s gene dosage on another feature’s gene expression levels cannot reasonably be assumed. This problem may be circumvented by limiting the matching to a set of DNA copy number features in the vicinity of the gene’s midpoint. Care should still be exercised. The Overlap matching procedure described later overcomes this problem.

### DistanceAny

**Figure 3:**
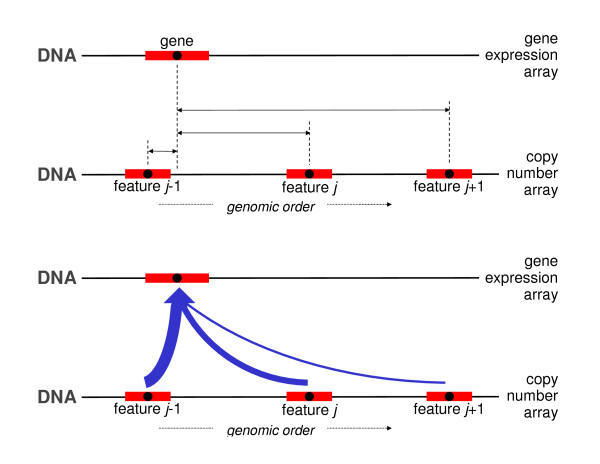
**Distance Any matching: the DNA copy number data of features (mapping to the same chromosome as the gene) is averaged with weights reciprocal to their distance to the gene.** In the top panel the distances between the features’ and the gene’s midpoints are represented by the horizontal solid arrows. In the bottom panel, the features’ DNA copy number data are averaged, the width of the arrows reflect the weights (reciprocal to the distances) of each feature’s contribution to the average.

Several features may be comparably close to the gene to be matched. The distance matching procedure however works in accordance to the winner-takes-all principle: the closest, even though only marginally closer than the runner-up, is assigned to the gene. This resembles the philosophy of a greedy algorithm. A more democratic approach would allow all features (not only one) to contribute, possibly in various degrees, to the matching (Figure 3). The distanceAny approach does exactly this, and takes into account the runners-up. Hereto, the distanceAny matching procedure assigns a weighted average of the DNA copy number features to the gene. When running over the genome, this is similar in spirit to a moving average. Weights may be chosen reciprocal to the distance, and possibly be limited to a neighborhood of the gene. The details of the distanceAny algorithm are contained in Table 3.

**Table 3:** DistanceAny matching

*Step 1*
For all genes, calculate their midpoints (average of start and end base pair position). Do the same for the features on DNA copy number array.
*Step 2*
For each gene, calculate the distance between its midpoint and that of the DNA copy number array features (mapping to the same chromosome as the gene). See the top panel of Figure 3.
*Step 3*
For each gene, calculate the weighted average of the DNA copy number data of the features (mapping to the same chromosome as the gene). Weights are chosen reciprocal to the distance of the features’ midpoint to that of the gene (bottom panel of Figure 3).

The possible disadvantages of the distance method directly transfer to the distance Any method. In particular, if the genome is highly unstable (exhibiting many breakpoints) at or near the location of the gene, the DNA copy number data assigned by the distanceAny procedure need not resemble the ‘true’ gene dosage of the gene.

### Overlap

**Figure 4:**
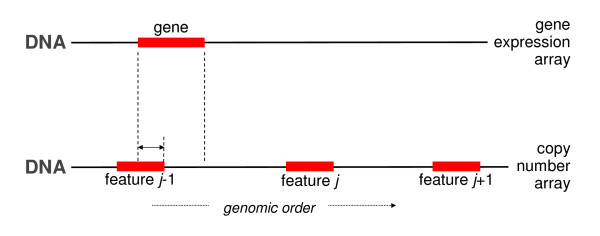
**Overlap matching: the DNA copy number feature with the maximum percentage of overlap in sequence with the gene is sought.** Here feature *j-1* overlaps with the gene (indicated by the horizontal solid arrow), whereas features *j* and *j* + 1 do not.

Instead of the distance between two features, the percentage of overlap between them may be employed to match the features from two platforms. A gene is matched to that DNA copy number feature with which it has the highest percentage of overlap. Among others De Menezes *et al*. [14] have used this approach. Table 4 describes the steps of the approach, while Figure 4 visualizes the key problem.

**Table 4:** Overlap matching

*Step 1*
For each gene, calculate the percentage of overlap between its sequence and that of the DNA copy number array features (mapping to the same chromosome as the gene). The percentage of overlap is defined as the number of overlapping base pairs between the two sequences divided by the length of the DNA copy number probe.
*Step 2*
Assign to each gene the DNA copy number data of the feature with the maximum percentage of overlap between its sequence and that of the gene.

The overlap procedure may be considered rather conservative (matching too few probes). This could be due to the fact that the features of both platforms may have a rather disjunct coverage of the genome. There may be valid biological grounds for this. But this disjunct coverage may also cause relatively few genes to be assigned a DNA copy number feature. The OverlapPlus approach aims to tackle this.

### OverlapAny

**Figure 5:**
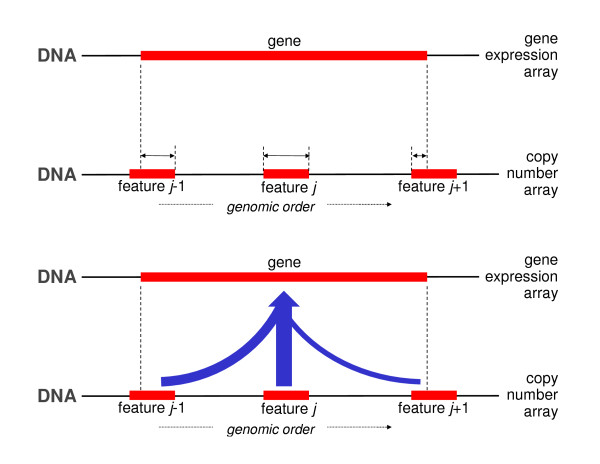
**OverlapAny matching: the DNA copy number data of features (mapping to the same chromosome as the gene) is averaged with weights proportional to their percentage of overlap with the gene’s sequence.** In the top panel the percentage of overlap between the features’ and the gene’s are represented by the horizontal solid arrows. In the bottom panel, the features’ DNA copy number data are averaged, the width of the arrows reflect the weights (proportional to the percentage of overlap) of each feature’s contribution to the average.

A gene may span a genomic region that is interrogated by multiple DNA copy number features. The overlap matching procedure then chooses an arbitrary feature that has its DNA copy number data assigned to the gene. Potentially relevant information on the DNA copy number of the gene is then ignored. Following the distanceAny matching approach, the data of all features with some overlap to the gene’s sequence is taken into account (via a weighting scheme) by the overlapAny approach (Figure 5). Contrasting the distanceAny method, the weights are proportional to the features’ percentage of overlap. Table 5 describes the steps of the overlapAny algorithm.

**Table 5:** OverlapAny matching

*Step 1*
For each gene, calculate the percentage of overlap between its sequence and that of the DNA copy number array features (mapping to the same chromosome as the gene). The percentage of overlap is defined as the number of overlapping base pairs between the two sequences divided by the length of the DNA copy number probe. See the top panel of Figure 5 for an illustration.
*Step 2*
For each gene, calculate the weighted average of the DNA copy number data of the features (mapping to the same chromosome as the gene). Weights are chosen proportional to the percentage of overlap of the features’ sequence to that of the gene. This is depicted in the bottom panel of Figure 5.

The disadvantages of the overlap matching procedure translate directly to the overlapAny approach.

### OverlapPlus

**Figure 6:**
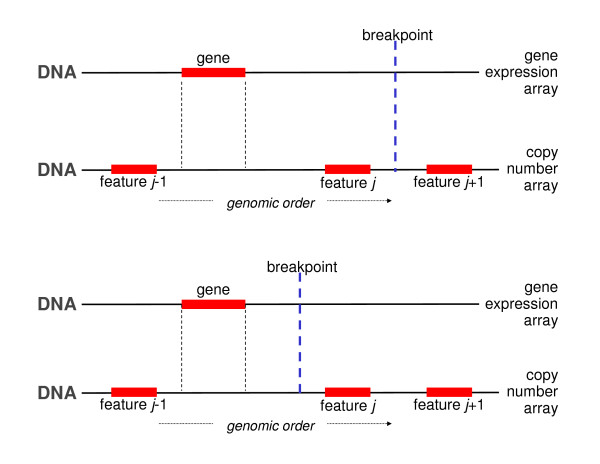
**OverlapPlus matching: after the overlap approach, the DNA copy number of unmatched genes is ‘estimated’ by interpolation of the gene dosage of the closest array CGH features.** In both panels no feature overlaps with the gene. In the top panel the overlapPlus approach would interpolate the DNA copy number data between feature *j-1* and *j*, as there is no breakpoint between them. In the bottom panel, however, the features *j-1* and *j* are separated by a breakpoint, and the overlapPlus procedure will not interpolate.

As the name suggests the overlapPlus matching procedure extends the overlap approach. Hereto overlapPlus alters the objective of feature matching. No longer are features of both platforms to be matched. Instead the new aim is to assign to each gene on the expression array the correct corresponding DNA copy number. This is achieved by first applying the overlap matching procedure. Then, DNA copy number information is interpolated to genomic areas not covered by the DNA copy number platform in order to assign to genes that map to these uncovered regions an “estimate” of their gene dosage (Figure 6). The interpolation is warranted by the discrete nature of the underlying biological phenomenon. This interpolation principle has (among others) been proposed by Autio *et al*. [13]. Table 6 details the steps of the overlapPlus algorithm.

**Table 6:** OverlapPlus matching

*Step 1*
Apply the overlap matching procedure.
*Step 2*
For all unmatched genes, the closest DNA copy number feature down- and upstream are
determined.
*Step 3*
Per gene, assess whether any of the samples in the dataset exhibits a breakpoint (i.e., a change in the segmented DNA copy number) in between the genes closest down- and upstream feature. If no breakpoint is present (the top panel of Figure 6), assign the DNA copy number data of the closest feature to the gene. If, any of the samples reveals a breakpoint (the bottom panel of Figure 6), the gene is left unmatched. For it could not be decided whether the closest down- or upstream feature contains the correct DNA copy number data.

A drawback of the overlapPlus approach is the fact that it uses, next to the feature annotation information, the experimental data (to assess the presence of a breakpoint). This makes the resulting matching dataset dependent: the matching may be different for subsets of the dataset.

### Extensions to distanceAny and overlapAny

The sigaR-package offers three extensions to the distanceAny and overlapAny procedures. These extensions concern the case where multiple DNA copy number features match to a gene, and distanceAny and overlapAny then take a weighted average. Instead of averaging, the first extension selects (in line with the ACEit-package [11]) the most extreme (operationalized as the largest absolute deviation from zero) segmented DNA copy number signal. This is done per sample individually. Consequently, the resulting DNA copy number signature may comprise data from all matched features. Selection of the ‘most aberrant signature’ leads to more variation in DNA copy number data, which may benefit the discovery of a *cis*-effect. However, this approach may also increase the chance of a false discovery.

The second extension encompasses the introduction of an additional step prior to the weighted average. It may happen that one or more of the samples exhibit(s) a breakpoint (a change in the segmented values) within the set of matched DNA copy number features. This extension splits the set of matched features at all breakpoints occurring within it in any of the samples, before proceeding to the weighted average. As a result, within the matched DNA copy number - gene expression data set, a gene may appear multiple times: each time with the same expression signature (the vector of expression values of that gene over the samples), but with a different DNA copy number signature (the vector of DNA copy numbers over the samples). When interested in *cis*-effect detection via univariate (gene-wise) analysis, this may increase the multiple testing correction.

The third and last modification extends the previous one. Now the set of matched DNA copy number features is split into a collection of sets, each containing only a single DNA copy number feature. Again, in the final matched data set the same gene maybe present multiple times. The multiplicity correction may further increase compared to the previous modifications.

These extensions do not affect the actual assignment of DNA copy number features to genes, but produce only minor changes to the DNA copy number data summary, and are thus not taken along in the remainder. Note that the results of next Section (and in particular Tables 7 and 8), which do not involve the summary of the data, do apply to the extensions.

**Table 7:** Matching results for the data sets using various matching procedures

	**Chin**	**TCGA I**	**TCGA II**	**Taylor I**	**Taylor II**
*platform details*					
manufacturer CN	UCSF	Agilent	Agilent	Agilent	Agilent
probe type CN	BAC	oligo	oligo	oligo	oligo
manufacturer GE	Affymetrix	Affymetrix	Agilent	Agilent	Affymetrix
probe type GE	oligo	oligo	oligio	oligo	oligo
*before matching*					
# samples	89	55	55	49	49
# CN features	2149	234416	234416	223697	223697
# GE features	10757	18528	35582	393	15478
*after* distance *matching*					
# CN and GE features	10757	18528	35582	393	15478
*after* distanceAny *matching* (< 10000 bp)					
# CN and GE features	190	18179	34620	389	15254
# CN features per gene (average)	1.04	2.92	2.88	4.36	2.87
*after* distanceAny *matching* (< 100000 bp)					
# CN and GE features	1921	18480	35424	393	15468
# CN features per gene (average)	1.13	24.33	23.84	27.97	24.47
*after* overlap *matching*					
# CN and GE features	1734	17426	32135	7	14900
*after* overlapAny *matching* (> 0%)					
# CN and GE features	1734	17426	32135	7	14900
# CN features per gene (average)	1.13	8.91	8.63	1	9.86
*after* overlapAny *matching* (> 0.10%)					
# CN and GE features	1585	17424	32123	5	14898
# CN features per gene (average)	1.10	8.91	8.63	1	9.85
*after* overlapPlus *matching*					
# CN and GE features	1879	18405	35197	349	15381

For the distanceAny and overlapAny the average number of DNA copy number features that contribute to the composition of the final DNA copy number signature is reported. This is one for all other methods.

**Table 8:** Matching results for the A_23_P140170 gene expression feature of the TCGA II data set

***Gene expression feature to be matched***									
Chr.	Start	End							
14	38570874	38642188							
*Matching DNA copy number features*									
Chr.	Start	End	dist.	dist.Any	dist.Any	overl.	over.Any	over.Any	over.Plus
				(< 10 k)	(< 100 k)		(> 0%)	(> 10%)	
14	38510003	38510062			x				
14	38542068	38542127			x				
14	38555801	38555860			x				
14	38562310	38562358			x				
14	38571564	38571623			x	x	x	x	x
14	38581765	38581824			x		x	x	
14	38587626	38587685			x		x	x	
14	38597108	38597167		x	x		x	x	
14	38602277	38602335		x	x		x	x	
14	38609230	38609289	x	x	x		x	x	
14	38614898	38614957		x	x		x	x	
14	38626492	38626551			x		x	x	
14	38631536	38631595			x		x	x	
14	38639169	38639225			x		x	x	
14	38646487	38646546			x				
14	38653819	38653878			x				
14	38665516	38665575			x				
14	38670657	38670716			x				
14	38675867	38675926			x				
14	38682480	38682539			x				
14	38686855	38686914			x				
14	38693139	38693198			x				
14	38697285	38697344			x				
14	38701438	38701497			x				
14	38705250	38705309			x				

An ‘x’ denotes that that DNA copy number feature has been selected by the corresponding matching procedure. The dashed lines indicate the boundaries of the gene.

## Results

Five data sets have been downloaded to compare the matching procedures. Data set 1, referred to as the Chin data set [29], is a study involving breast cancer samples with genome and transcriptome profiled. Data sets 2 and 3, referred to as the TCGA I and II data sets (respectively), comprise glioblastoma data from The Cancer Genome Atlas, both with DNA copy number and gene expression data available for all samples. The TCGA I and II data sets differ in their gene expression data, which have been generated on different platforms. Data sets 4 and 5, referred to as the Taylor I and II data sets (respectively), consist of prostate cancer samples with DNA copy number and microRNA expression profile (Taylor I) and DNA copy number and exon expression profile (Taylor II). The DNA copy number data of both data sets (Taylor I and II) are identical. Details on the data sets are found in Table 7 (e.g., number of samples, number of features), and more extensively in Appendix A (including preprocessing details).

### Matching

Features of the platform pairs that produced the five data sets are matched by the following procedures: distance, distanceAny, overlap, overlapAny, and overlapPlus. Note the label-procedure is not taken along, for it is not applicable to the Chin and Taylor I data sets (there will be no matching as labels of BACs, the DNA copy number probes of the Chin data set, and microRNA probes need not map to a gene label).

The results of application of the matching procedures as implemented in the sigaR-package to the five data sets are presented in Table 7. In all data sets the distance procedure is most successful in terms of the number of gene expression features that have been matched to a DNA copy number feature: it has been able to match every gene to a DNA copy number feature (100% matching). This comes as no surprise, as the result would be observed even if a gene’s chromosome was interrogated by only one element of the array CGH. This ‘success’, however, comes at a price: some genes are matched to DNA copy number features far away from the genes’ location. The distanceAny procedure resolves this drawback by limiting the search for a matching DNA copy number feature to a subdomain of the genome. For the TCGA I and II and Taylor I and II data sets this yields over 97% matching. However, the number of matched gene expression features falls dramatically (to 17.9%) for the Chin data set, even when using a rather wide search window (100000 bp from the midpoint in both directions). This need not be a concern as the Chin DNA copy number data were generated on an BAC platform, which is being phased out. The even worse ‘performance’ (1.8% matched gene expression features) on the Chin data set of the distanceAny procedure with a smaller window may be attributed to the size of DNA copy number features (BACs). They are rather long compared to the gene expression features, resulting in distances between the midpoints of features from both platforms that often exceed the threshold of 10000 bp. As an alternative to the distanceAny procedure, one may use the overlap or overlapAny procedure to circumvent its drawbacks. For the TCGA I and II data sets, the percentage of matched gene expression features (all over 90%) are not far behind that of the distance and distanceAny procedures, and they exhibit a comparable ‘performance’ for the Chin and Taylor II data sets (16.1% and 96.2%, respectively) data as the distanceAny procedure. But they perform poorly (1.8%) for the Taylor I data set. This is due to the fact that there simply are no more overlapping features between the two platforms. The explanation may be two-fold: 1) microRNAs are much smaller than mRNAs, and 2) DNA copy number features present on the array may be undersampled at the location of microRNAs. A relaxation of the nonzero overlap between features from both platforms is offered by the overlapPlus procedure. This works out nicely for the Taylor I data set (the percentage of matched gene expression features now at 88.8%), and slightly improves the number of matched features for the other data sets. However, the overlapPlus procedure makes use of the experimental data (breakpoints), which implies that the matching may be different between data sets generated on the same platform.

As seen from the above, the distanceAny and overlapAny procedures come with a tuning parameter (the separation distance and the percentage of overlap, respectively), which affects the number of matched features. Not directly obvious, but no less important, the tuning parameter also determines the total number of DNA copy number features used in the construction of the matched gene dosage signature (the vector of DNA copy number values of one genomic location over samples). Whereas distance, overlap and overlapPlus eventually select a single feature from the DNA copy number array, the distanceAny and overlapAny procedures potentially select more than one feature, and their data is aggregated into a matching signature. Hence, the latter two procedures make more use of the experimental data.

### Consequences

To contrast these high-level comparisons of the matching procedures, we show the consequences of employing a particular matching procedure at the level of an individual gene. Table 8 shows the resulting matchings for a single gene of the TCGA II data set. It becomes quickly obvious that the matched DNA copy number features differ in number across matching procedures. More interesting is perhaps how the coverage of the gene varies between these sets of matched DNA copy number features. The distance matching procedure selects a single DNA copy number feature close to the middle of the gene. The distanceAny (< 10000 bp) procedure covers a larger interval around the midpoint of the gene, but stays well within the 3′ and 5′ ends of the gene. With a larger window size (< 100000 bp), the distanceAny procedure spans a region well beyond the boundaries of the gene. In contrast, the overlapAny procedures yield a reasonably uniform coverage of the gene not exceeding the 3′ and 5′ ends. Completely different in their choice of the matching DNA copy number feature are the overlap and overlapPlus procedures, which select the feature closest to the 3′ end of the gene. Too large a coverage, e.g., beyond the limits of the gene, may lead to a DNA copy number signature unrelated to the gene. Too small a coverage may assign it a rather noisy DNA copy number signature. The middle ground, with a reasonable coverage of the gene as shown by the overlapAny and distanceAny (with a small window size) procedures, seems an acceptable compromise.

**Figure 7:**
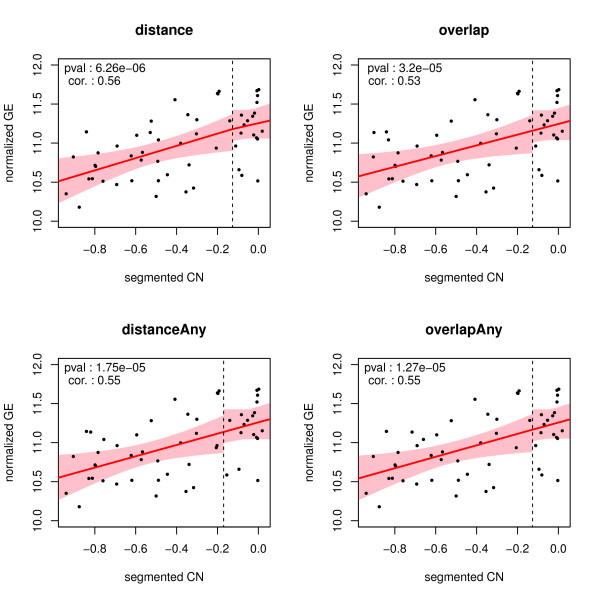
**Each panel depicts the relation between the matched gene expression and DNA copy number data (as produced by one of the matching procedure) for the 210774_s_at probe from the TCGA data set (with Affymetrix expression arrays).** The red line is the best fitting piece-wise linear spline (as obtained from the method described in [30]). The pink area represent the 95% confidence intervals for the fitted relationship. The vertical dashed line separate the samples with a loss from those with a normals.

**Figure 8:**
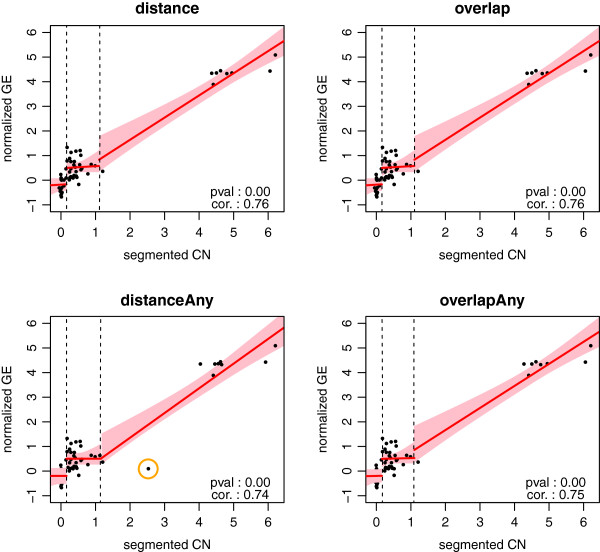
**Each panel depicts the relation between the matched gene expression and DNA copy number data (as produced by one of the matching procedure) for the A_23_P168211 probe from the TCGA data set (with Agilent expression arrays).** The red line is the best fitting piece-wise linear spline (as obtained from the method described in [30]). The pink area represent the 95% confidence intervals for the fitted relationship. The vertical dashed lines separate the samples with a normal from those with a gain, and those with a gain from those with an amplification.

The consequences of choosing a matching procedure reveal themselves also in DNA copy number data, as matching procedures either select different features or utilize different ways of summarizing data from multiple features. The vast majority of genes have DNA copy number signatures that vary little to nothing between the matching procedures (Figure 7). As a result, the *p*-values and Spearman’s rank correlations differ too, but again little. Occasionally, however, there is a data point that is affected in a more serious manner by the choice of matching procedure. Figure 8 shows that the distanceAny method has one data point (indicated by the orange circle) that deviates from its counterpart in the other matched DNA copy number signatures. In this particular case, it is due to the large window size chosen, and the problem vanishes if the window size is decreased.

One expects that the correlation between a gene’s expression levels and its true DNA copy number signature is higher than that between the gene’s expression levels and any other DNA copy number signature. This suggests that the best matching procedure yields the highest correlations between the two molecular levels. Therefore, for the genes present in all matched versions of a data set, we calculated the Spearman’s rank correlation coefficient between the genes’ expression levels and their assigned DNA copy number signature (segmented data). For many genes, the matching procedures yield identical correlations. Even when focussing on those genes with correlations varying over the matching procedures, the differences are often small. To provide some insight in which procedure yields the highest correlations, we compare the correlations of the matching procedures in a pairwise fashion. Hereto we simply count how many times matching procedure A yields a higher correlation than matching procedure B, and vice versa. Table 9 gives the results for the Chin and TCGA II data sets. For the Chin data set, the distance and distanceAny methods give the best results (more correlations that exceed that of other procedures than vice versa). E.g., the distance procedure yields 8 − 1 = 7 genes with a higher *cis*-correlation than the overlapPlus procedure. The distance and distanceAny methods are followed by the overlapAny procedure, and finally, but not too far behind, the overlap and overlapPlus procedures. A similar picture emerges from the Taylor II data set (results not shown). The TCGA I (results not shown) and TCGA II data sets tell a different story: the overlapAny procedure performs best, followed by the distanceAny with a small window. No clear winner emerges from this comparison, but it points to either the distanceAny (with a small window size) or overlapAny procedure. Or, put differently, in the light of the results presented in Table 8, this points to procedures capable of matching multiple DNA copy number features to the same gene, and that together have a reasonable coverage of that gene.

**Table 9:** **Pairwise comparison of *cis*-correlations**

	**dist.**	**dist.Any**	**dist.Any**	**overl.**	**over.Any**	**over.Any**	**over.Plus**
		**(< 10 k)**	**(< 100 k)**		**(> 0%)**	**(> 10%)**	
distance							
Chin	-	15	7	8	15	14	8
TCGA II	-	5869	778	1253	1632	1641	1253
distanceAny (< 10 k)							
Chin	1	-	10	17	15	15	17
TCGA II	919	-	5957	6153	5694	5698	6153
distanceAny (< 100 k)							
Chin	11	9	-	9	11	10	9
TCGA II	6102	5821	-	1808	1812	1823	1808
overlap							
Chin	1	9	4	-	8	7	0
TCGA II	1294	6090	1770	-	1883	1885	0
overlapAny (> 0%)							
Chin	10	12	14	13	-	5	13
TCGA II	2071	5807	2131	2116	-	168	2116
overlapAny (> 10%)							
Chin	10	12	14	11	3	-	11
TCGA II	2074	5810	2137	2122	161	-	2122
overlapPlus							
Chin	1	9	4	0	8	7	-
TCGA II	1294	6090	1770	0	1883	1885	-

The upper triangle contains the number of genes (within the set of intersecting genes) with *cis*-correlations (Spearman) of the column matching procedure exceeding those of the row matching procedure. Low triangle similar, but defined vice versa. Recall the number of intersecting genes equals 157 (Chin) and 31947 (TCGA II).

### Downstream analysis

**Figure 9:**
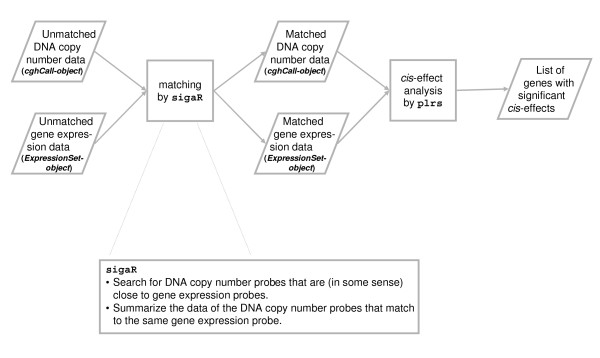
**Flowchart of *cis*-effect analysis.**

Finally, we illustrate the effect of matching on downstream analysis. We assess the *cis*-effect of a DNA copy number aberration on the expression levels of the genes mapping to it. Hereto, we employ piecewise linear regression splines (PLRS) to allow any plausible type of relationship between the two molecular levels [Leday GGR, Van der Vaart AW, Van Wieringen WN, Van de Wiel MA (2012) “Modeling association between DNA copy number and gene expression with constrained piecewise linear regression splines”, *submitted*; 31]. A gene’s association between gene dosage and expression levels is declared significant if its corrected *p*-value (Benjamini-Hochberg multiple testing correction) is smaller than 0.05. The associated workflow is portrayed in Figure 9. Table 10 reports the number of significant genes for each data set - matching procedure combination (the Taylor I and II data sets are excluded for being uninformative, neither provided anything significant). This number is reported on the whole set of genes matched by each procedure (the size of this set can be found in Table 7), but also on the restricted set containing only those genes that are matched by all procedures. The Taylor I and Taylor II data sets are not discriminative between the matching procedures. For the Chin data set the distance procedure finds most significant genes, followed by distanceAny (< 100 k), overlapPlus and other overlap methods. This order is concordant with the matching result: the more matched genes, the more discoveries. This may obscure the comparison of the methods. Moreover, as pointed out before, the distance and distanceAny (with a large window size) procedures may match genes to DNA copy number features located elsewhere on the genome. This raises doubts over the interpretation of significant associations. In the restricted set of genes, the number of discoveries is constant over the methods, with the overlapAny procedure having one additional finding. In the TCGA I and TCGA II data sets, irrespective of the gene sets considered, the overlapAny procedures yield most significant findings. Noteworthy is the fact that even though the distance-based procedures match hundreds (TCGA I) or thousands (TCGA II) of genes more, this does not lead to more discoveries. This could be interpreted as the additionally matched genes being assigned an unrelated DNA copy number signature. In summary, this comparison of downstream analyses suggests that (at least in data sets generated on a high-resolution DNA copy number platform) the overlapAny procedure may be preferred.

**Table 10:** Results of downstream analysis

	**Chin**	**TCGA I**	**TCGA II**
	**(157)**	**(17352)**	**(31947)**
distance *matching*			
individual	4009	929	9845
intersection	66	913	9768
distanceAny *matching* (< 10000 bp)			
individual	74	914	9772
intersection	66	932	9769
distanceAny *matching* (< 100000 bp)			
individual	787	937	9814
intersection	66	924	9740
overlap *matching*			
individual	675	912	9824
intersection	66	911	9801
overlapAny *matching* (> 0%)			
individual	684	937	9810
intersection	67	935	9779
overlapAny *matching* (> 0.10%)			
individual	640	937	9809
intersection	67	935	9778
overlapPlus *matching*			
individual	737	920	9859
intersection	66	909	9800

Number of genes with a significant *cis*-effect at *FDR* < 0.05 for the data set-matching combinations. The number of genes is displayed for data sets as yielded by each matching procedure individually, and for the data sets confined to the set of intersecting genes. The total number of intersecting genes is given directly below to the data set name.

## Conclusion

Matching of the features from different high-throughput platforms is a important preprocessing step for bioinformatic analyses of integrative genomics studies. We have described, reviewed and implemented (sigaR-package) the most widely used matching procedures found in literature. Application of the matching procedures to five data sets generated on different platforms revealed that 1) the number of features matched varies considerably between the matching procedures, and 2) the choice of matching procedure may even affect (although usually only to a minor degree) the DNA copy number signature (the vector of DNA copy number values over the samples) assigned to a gene. These observations, which have their consequences on any downstream integrative analysis, facilitate an informed decision on the matching procedure of choice.

The matching procedures have shown little difference in the number of features matched and have very little impact on downstream analysis results, in the several examples shown. These results rely on correct pre-processing, of which copy number data segmentation is an important aspect. It should be kept in mind that, although overall results may be robust to matching procedure selection, this may not be true for all genes, as Figure 8 illustrates. Finally, care should be exercised when extrapolating these conclusions to other data sets, which may have been generated using other platforms/technologies than those used here.

We recommend to start the matching with the overlapAny procedure. This may be conservative in some cases, but certainly has the clearest and most undisputed physical interpretation for matching. If this yields satisfactory results, the task is done. Else, remaining unmatched features may be handled either by the distanceAny (with not too big a window around the gene) or overlapPlus procedure.

## Availability and requirements

• Project name: sigaR

• Project home page: http://www.bioconductor.org

• Operating system: Platform independent

• Programming language: R

• Other requirements: R (currently: R-devel; soon: >= 2.15.0)

• License: General Public Licence (>= 2)

• Any restrictions to use by non-academics: none declared.

## Appendix A: Data sets

Data set 1: Chin

• *Sample type:* Breast cancer.

• *Molecular levels:* DNA copy number & gene expression.

• *Reference:* Chin *et al.*[29].

• *DNA copy number platform:* BAC, fabricated at UC San Francisco.

• *Gene expression platform:* Affymetrix U133A.

• *Number of samples:* 89.

• *Availability:* CaBig repository.

• *Preprocessing:* Pre-processing of both DNA copy number and gene expression data used here was as described in [32], with the additional steps of segmentation and calling (via the R-package CGHcall [33], using default settings) on the normalized data. The annotation information of both datasets was updated as described below. The publicly available DNA copy number data had an annotation table involving chromosome number, start and end positions, with the latter equal to exactly the start plus 2 bp, for all BAC clones. As this is unlikely to be true and correct information is essential for matching to be performed adequately, annotation information for BAC clones from Ensembl was used to update the information. For 1491 BAC clones in the Chin data, we obtained updated start and end positions. For the remaining clones, not found via Ensembl, their chromosome and start data were kept the same, but their end location was imputed by the sum of their start plus the average BAC clone length on the newer annotation table (144132 bp). The Chin gene expression array data contained 21339 probe sets. Using the Bioconductor package hgu133plus2.db version 2.4.1, we obtained up-to-date annotation (including start and end chromosomal positions) for 16099 probe sets. Some of those were allocated to more than one chromosome, in which case we took the first values for chromosome, start and end encountered in the data table.

Data set 2: TCGA I

• *Sample type:* Glioblastoma.

• *Molecular levels:* DNA copy number & gene expression.

• *Reference:* Verhaak *et al. *[34].

• *DNA copy number platform:* 244 K Agilent MSKCC.

• *Gene expression platform:* Affymetrix 133A.

• *Number of samples:* 55.

• *Availability:* The Cancer Genome Atlas (TCGA): http://cancergenome.nih.gov/

• *Preprocessing:* All samples from batch 1 to 3, for which both copy number data (244 K Agilent MSKCC) and level-e normalized expression data (Affy 133A) is available, were used. Replicated samples were not taken into account. Probe features were matched to genomic locations using Ensembl and biomaRt (NCBI build 36, Ensemble 54). The Affymetrix gene expression array contains 22277 probe features, of which 18607 could be matched to a genomic location. After removal of probes mapping to the Y chromosome, 18528 probes for the gene expression data were left. The Agilent copy number platform consists of 235834 probe features of which all features could be matched to a genomic location. 234416 were available after preprocessing in CGHcall [33]. Segmentation and calling were performed using default settings except for undo.sd (0.6).

• *Note:* The DNA copy number data of this data set is identical to that of the TCGA II data set.

Data set 3: TCGA II

• *Sample type:* Glioblastoma.

• *Molecular levels:* DNA copy number & gene expression.

• *Reference:* Verhaak *et al.*[34].

• *DNA copy number platform:* 244 K Agilent MSKCC.

• *Gene expression platform:* Agilent custom 244 K.

• *Number of samples:* 55.

• *Availability:* The Cancer Genome Atlas (TCGA): http://cancergenome.nih.gov/

· *Preprocessing:* All samples from batch 1 to 3, for which both copy number data (244K Agilent MSKCC) and level-2 normalized expression data (Agilent custom 244K) is available, were used. Replicated samples were not taken into account. Probe features were matched to genomic locations using Ensembl and biomaRt (NCBI build 36, Ensemble 54). The Agilent gene expression array is a custom design on the 244K platform with 90K unique probes. Only 36196 of the 90797 unique probes could be matched to genomic locations using Ensembl. After removal of probes mapping to the Y chromosome, 35582 probes for the gene expression data were left. The Agilent copy number platform consists of 235834 probe features of which all features could be matched to a genomic location. 234416 were available after preprocessing in CGHcall [33]. Segmentation and calling were performed using default settings except for undo.sd (0.6).

• *Note:* The DNA copy number data of this data set is identical to that of the TCGA I data set.

Data set 4: Taylor I

• *Sample type:* Prostate cancer.

• *Molecular levels:* DNA copy number & microRNA expression.

• *Reference:* Taylor *et al.*[35].

• *DNA copy number platform:* Agilent 244 K array CGH.

• *MicroRNA expression platform:* Agilent microRNA V2.

• *Number of samples:* 49.

• *Availability:* GEO database (DNA copy number: GSE21034; Exon expression: GSE21036).

• *Preprocessing:* Data are limited to 49 prostate cancer samples of which DNA copy number, exon expression and microRNA expression (see data set Taylor I) was available. The Agilent 244 k array CGH raw data were provided as tab-delimitedtext files containing the normalised *log*_2_-ratios and the values of control measures such as background-to-foreground ratios, spot saturation, spot uniformity and others. The files were imported into R and the data filtered for the aforementioned quality criteria and subsequently merged with the recent annotation data provided by Agilent eArray. The data was segmented and called using the CGHcall-package [33] using default settings. The resulting data consists of 223697 oligo probes (autosomes only). The Agilent Human miRNA Microarray 2.0 (GEO platform GPL8227) data were downloaded as text files and imported and processed in R using the Bioconductor package AgiMicroRna [36]. The *log*_2_ expression values were normalized using RMA [37] and the resulting data matrix filtered for QA criteria provided by Agilent that make sure that only meaningful expression values are kept in the dataset. The final data object contained the expression values of 393 miRNAs (autosomes only) from 49 samples.

• *Note:* The DNA copy number data of this data set is identical to that of the Taylor II data set.

Data set 5: Taylor II

• *Sample type:* Prostate cancer.

• *Molecular levels:* DNA copy number & exon expression.

• *Reference:* Taylor *et al.*[35].

• *DNA copy number platform:* Agilent 244 K array CGH.

• *Exon expression platform:* Affymetrix Human Exon 1.0 ST array.

• *Number of samples:* 49.

• *Availability:* GEO database (DNA copy number: GSE21034; Exon expression: GSE21036).

• *Preprocessing:* Data are limited to 49 prostate cancer samples of which DNA copy number, exon expression and microRNA expression (see data set Taylor I) was available. The Agilent 244 k array CGH raw data were provided as tab-delimitedtext files containing the normalised *log*_2_-ratios and the values of control measures such as background-to-foreground ratios, spot saturation, spot uniformity and others. The files were imported into R and the data filtered for the aforementioned quality criteria and subsequently merged with the recent annotation data provided by Agilent eArray. The data was segmented and called using the CGHcall-package [33] using default settings. The resulting data consists of 223697 oligo probes (autosomes only). Preprocessing of the Affymetrix Human Exon 1.0 ST Array data (GEO platform GPL5188) followed the workflow described in [38]. Briefly, Affymetrix Power Tools was used to read the raw data CEL files with hybridisation fluorescence intensities along with the latest version of annotation files and to normalise the gene-level data using the Robust Multichip Average (RMA) algorithm [37]. After quality filtering the result is stored in an ExpressionSet-object with summarized *log*_2_-ratios of 15478 genes (autosomes only) from 49 samples.

· *Note:* The DNA copy number data of this data set is identical to that of the Taylor I data set.

## Appendix B: Example code

For the R-code below it is assumed that the sigaR-package plus its dependencies have been activated, and that the cghCall and ExpressionSet objects (called CNdata and GEdata, respectively) have been loaded (Tables 11, 12, 13, 14, 15, 16, 17 and 18).

**Table 11:** R-code for distance matching

**# match**
> matchedIDs < − matchCGHcall2ExpressionSet(CNdata, GEdata, 1, 2, 3,
1, 2, 3, method = “distance”)
> # generate matched objects
> CNdata < − cghCall2subset(CNdata, matchedIDs[,1])
> GEdata < − ExpressionSet2subset(GEdata, matchedIDs[,2])

**Table 12:** R-code for overlap matching

**# match**
> matchedIDs < − matchCGHcall2ExpressionSet(CNdata, GEdata, 1, 2, 3,
1, 2, 3, method = “overlap”)
> # generate matched objects
> CNdata < − cghCall2subset(CNdata, matchedIDs[,1])
> GEdata < − ExpressionSet2subset(GEdata, matchedIDs[,2])

**Table 13:** R-code for overlapPlus matching

**# match**
> matchedIDs < − matchCGHcall2ExpressionSet(CNdata, GEdata, 1, 2, 3,
1, 2, 3, method = “overlapPlus”)
> # generate matched objects
> CNdata < − cghCall2subset(CNdata, matchedIDs[,1])
> GEdata < − ExpressionSet2subset(GEdata, matchedIDs[,2])

**Table 14:** R-code for distanceAny matching

**# match**
> matchedIDs < − matchAnn2Ann(fData(CNdata)[,1], fData(CNdata)[,2],
fData(CNdata)[,3], fData(GEdata)[,1],
fData(GEdata)[,2], fData(GEdata)[,3],
method = “distance”)
> # add offset to distances (avoids infinitely large weights)
> matchedIDs < − lapply(matchedIDs, function(Z, offset){ Z[,3] < − Z[,3] + offset;
return(Z) }, offset = 1)
> # extract ids for object subsetting
> matchedIDsGE < − lapply(matchedIDs, function(Z){ return(Z[, -2, drop=FALSE]) })
> matchedIDsCN < − lapply(matchedIDs, function(Z){ return(Z[, -1, drop=FALSE]) })
> # generate matched objects
> GEdata < − ExpressionSet2weightedSubset(GEdata, matchedIDsGE, 1, 2, 3)
> CNdata < − cghCall2weightedSubset(CNdata, matchedIDsCN, 1, 2, 3)

**Table 15:** R-code for overlapAny matching

**# match**
> matchedIDs < − matchAnn2Ann(fData(CNdata)[,1], fData(CNdata)[,2],
fData(CNdata)[,3], fData(GEdata)[,1],
fData(GEdata)[,2], fData(GEdata)[,3],
method = “overlap”)
> # extract ids for object subsetting
> matchedIDsGE < − lapply(matchedIDs, function(Z){ return(Z[, -2, drop=FALSE]) })
> matchedIDsCN < − lapply(matchedIDs, function(Z){ return(Z[, -1, drop=FALSE]) })
> # generate matched objects
> GEdata < − ExpressionSet2weightedSubset(GEdata, matchedIDsGE, 1, 2, 3)
> CNdata < − cghCall2weightedSubset(CNdata, matchedIDsCN, 1, 2, 3)

**Table 16:** R-code for overlapAny matching with extension 1: extreme DNA copy number signal

**# match**
> matchedIDs < − matchAnn2Ann(fData(CNdata)[,1], fData(CNdata)[,2],
fData(CNdata)[,3], fData(GEdata)[,1],
fData(GEdata)[,2], fData(GEdata)[,3],
method = “overlap”)
> # extract ids for object subsetting
> matchedIDsGE < − lapply(matchedIDs, function(Z){ return(Z[, -2, drop=FALSE]) })
> matchedIDsCN < − lapply(matchedIDs, function(Z){ return(Z[, -1, drop=FALSE]) })
> # generate matched objects
> GEdata < − ExpressionSet2weightedSubset(GEdata, matchedIDsGE, 1, 2, 3)
> CNdata < − cghCall2maximumSubset(CNdata, matchedIDsCN, 1, 2, 3)

**Table 17:** R-code for overlapAny matching with extension 2: split at breakpoints

**# match**
> matchedIDs < − matchAnn2Ann(fData(CNdata)[,1], fData(CNdata)[,2],
fData(CNdata)[,3], fData(GEdata)[,1],
method = “overlap”)
> # expand > matchedFeatures < − splitMatchingAtBreakpoints(matchedFeatures, CNdata)
> # extract ids for object subsetting
> matchedIDsGE < − lapply(matchedIDs, function(Z){ return(Z[, -2, drop=FALSE]) })
> matchedIDsCN < − lapply(matchedIDs, function(Z){ return(Z[, -1, drop=FALSE]) })
> # generate matched objects
> GEdata < − ExpressionSet2weightedSubset(GEdata, matchedIDsGE, 1, 2, 3)
> CNdata < − cghCall2weightedSubset(CNdata, matchedIDsCN, 1, 2, 3)

**Table 18:** R-code for overlapAny matching with extension 3: link gene to each matched DNA copy number feature seperately

**# match**
> matchedIDs < − matchAnn2Ann(fData(CNdata)[,1], fData(CNdata)[,2],
fData(CNdata)[,3], fData(GEdata)[,1],
fData(GEdata)[,2], fData(GEdata)[,3],
method = “overlap”)
> # expand > matchedIDs < − expandMatching2singleIDs(matchedIDs)
> # extract ids for object subsetting
> matchedIDsGE < − lapply(matchedIDs, function(Z){ return(Z[, -2, drop=FALSE]) })
> matchedIDsCN < − lapply(matchedIDs, function(Z){ return(Z[, -1, drop=FALSE]) })
> # generate matched objects
> GEdata < − ExpressionSet2weightedSubset(GEdata, matchedIDsGE, 1, 2, 3)
> CNdata < − cghCall2maximumSubset(CNdata, matchedIDsCN, 1, 2, 3)

## Competing interests

The authors declare that they have no competing interest.

## Authors contributions

WNvW conceived and carried out the project, and wrote the paper. KU, OK and RXdM provided the data. KU, GGRL, OK, RXdM, BY and MAvdW contributed to the development of at least one of the matching procedures, and made comments and suggestions. All authors read and approved of the manuscript.
